# Deterioration in the Quality of ‘Xuxiang’ Kiwifruit Pulp Caused by Frozen Storage: An Integrated Analysis Based on Phenotype, Color, Antioxidant Activity, and Flavor Compounds

**DOI:** 10.3390/foods14132322

**Published:** 2025-06-30

**Authors:** Chenxu Zhao, Junpeng Niu, Wei Wang, Yebo Wang, Linlin Cheng, Yonghong Meng, Yurong Guo, Shujie Song

**Affiliations:** 1 College of Food Engineering and Nutritional Science, Shaanxi Normal University, Xi’an 710119, China; zcx96429@126.com (C.Z.); ww12020412@163.com (W.W.); wangyebo0525@163.com (Y.W.); chenglinlin0619@163.com (L.C.); mengyonghong@snnu.edu.cn (Y.M.); 2 Engineering Research Center of High-Valued Utilization of Fruit Resources in Western China, Ministry of Education, Xi’an 710119, China; 3 College of Life Sciences, Shaanxi Normal University, Xi’an 710119, China; niujp100@163.com

**Keywords:** *Actinidia deliciosa*, frozen duration, chlorophyll, tackiness, vitamin C

## Abstract

Kiwifruit has attracted much attention in fruit and vegetable processing due to its high nutritional and economic value. However, there is a lack of systematic research on the effects of long-term frozen storage on the pulp quality of kiwifruit. Using kiwifruit pulp stored at −20 °C for 0, 3, 6, 9, and 12 months as the research materials, the dynamic changes in the phenotype, color, antioxidant activity, and flavor compounds were comprehensively evaluated. The results showed that frozen storage caused a significant decline in the quality of the fruit pulp. Specifically, the contents of chlorophyll and carotenoids decreased and the color deteriorated (color difference increased); the turbidity and centrifugal sedimentation rates increased, and pH and viscosity changed in different stages. Additionally, antioxidant compounds, such as vitamin C and total phenols, were significantly reduced with the extension of storage duration, and the 2,2-diphenyl-1-picrylhydrazyl (DPPH)/2,2-azino-bis (3-ethylbenzothiazoline-6-sulfonic acid) (ABTS) free radical scavenging ability was decreased. The content of volatile aroma compounds diminished, leading to a notable shift in the flavor profile. Correlation analysis revealed that changes in volatile substances were significantly correlated with physical, chemical, and antioxidant indicators (*p* < 0.05). These correlations can serve as a key basis for assessing quality deterioration. This study systematically elucidated, for the first time, the mechanism of quality deterioration in kiwifruit pulp during frozen storage, thereby providing theoretical support for enterprises to optimize pulp grading strategies and the timing of by-product development. Hence, it is recommended that the duration of freezing should be limited to less than 9 months for kiwifruit pulp. Moreover, it is essential to consider varietal differences and new pretreatment technologies to further enhance the industrial utilization and economic value of frozen pulp.

## 1. Introduction

Kiwifruit (*Actinidia* spp.) is rich in vitamin C (Vc), polyphenols, and antioxidant activity, and it holds significant economic value [1]. The methods of post-harvest storage and processing are currently hot topics in the food industry [2]. Freezing storage (−20 °C) is a primary technique used to maintain quality and extend shelf life of fruit and vegetable processing products [3,4]. This method can slow down quality deterioration by inhibiting enzyme activity and microbial growth [5,6]. However, there exists a lack of systematic research addressing issues such as appearance deterioration, oxidative stress, and the loss of flavor compounds caused by long-term freezing, which significantly limits the industrial utilization of fruit pulp [7].

Kiwifruit can be stored at 0 °C and 90–95% RH for several months by means of a controlled atmosphere containing 3 kPa oxygen and 5 kPa carbon dioxide [8]. However, there are still adverse effects, such as lignification of the fruit core and deterioration of the flesh color [9]. Therefore, processing kiwifruit into by-products, such as jam, can significantly enhance its economic value. Numerous studies have shown that freezing can effectively inhibit microorganisms and enzymatic reactions over extended periods, but the damage to cellular structures caused by ice crystal formation can still adversely affect the physical and chemical properties of fruit [10]. For instance, after 6 months of storage at −18 °C, the loss rate of Vc in apple reached 30% [5]. The dynamic changes in volatile organic compounds are significantly and negatively correlated with storage time, which was demonstrated by the half-life (6–8 months) of esters [11]. When peaches were stored below 0 °C, there was a marked reduction in volatile esters, leading to a deterioration in flavor [11]. In addition, temperature fluctuations can enhance the activity of cell-wall-degrading enzymes, resulting in fruit softening [8]. Even prolonged freezing can diminish the levels of polyphenols and flavonoids through oxidative stress, thereby affecting antioxidant capacity [12]. Extended freezing for more than 12 months may induce ice crystal recrystallization, increase cell membrane permeability, and accelerate irreversible damage [13]. Although there is emphasis on the synergistic effect of new pretreatment technologies (such as the application of the ethylene inhibitor 1-methycyclopropene (1-MCP) and the technical optimization of low-temperature warehouses) on kiwifruit storage in recent years [8], there remains a gap in the systematic evaluation of the multi-dimensional quality deterioration of kiwifruit pulp following long-term frozen storage in the industry.

Therefore, this study aimed to clarify the quality deterioration dynamics of kiwifruit pulp during frozen storage at −20 °C for 12 months. This was achieved by integrating multiple data types, including phenotype, physicochemical indicators, antioxidant ability, and flavor profiles. The research findings could offer a theoretical foundation for optimizing the processing parameters of frozen fruit pulp, establish a model for changes in key quality parameters, and guide enterprises in developing differentiated grading strategies for fruit pulp. This study addresses the gap in understanding the multifaceted impact of frozen storage on the fruit pulp matrix and holds significant practical implications for enhancing the economic benefits of the kiwifruit processing industry chain.

## 2. Materials and Methods

### 2.1. Fruit Materials

‘Xuxiang’ kiwifruit (*Actinidia deliciosa*) was harvested from an orchard in Zhouzhi County, Shaanxi Province. The fruit was uniform in size and maturity, free from pests and mechanical damage, with a dry matter content exceeding 15%. The freezing process for the kiwifruit was conducted according to the methods used in jam processing factories, employing rapid freezing at −40 °C to lower the core temperature to −20 °C, followed by immediate transfer to a −20 °C refrigerator for storage. In each batch, 60 fruits were tested at 3, 6, 9, and 12 months. The fruits were removed and placed under running water at room temperature to thaw [14]. Thawing was considered complete when all ice crystals had disappeared. At each testing interval, the fruits in each batch were divided into three groups, with 20 fruits randomly selected from each group. After peeling and pulping, the sample was briefly stored in a refrigerator at 4 °C for subsequent measurements.

### 2.2. Determination of Fruit Quality

#### 2.2.1. Determination of Color

Measurements of kiwifruit pulp (at 0.5 cm from the skin) and sarcocarp were carried out using a colorimeter (NH 310, Shenzhen, China) with a chromaticity value, hue angle value (h*), L* (luminance), a* (red/green), and b* (yellow/blue) [15]. Six fruits were measured in each group.

#### 2.2.2. Determination of Chlorophyll and Carotenoids

About 2 g of kiwifruit pulp was ground with 2–3 mL of 95% ethanol, including a small amount of SiO_2_ and CaCO_3_. The homogeneous mixture was leached with additional 95% ethanol and then filtered using filter paper. Chlorophyll a, chlorophyll b, chlorophyll, and carotenoids were determined using a spectrophotometer (Pulsar T9CS, Beijing, China) at 470, 645, and 663 nm, respectively; and they are calculated using Equations (1)–(3) [16,17].

(1) $$Ca=13.95\times A_{663}-6.88\times A_{645}$$

(2) $$Cb=24.96\times A_{645}-7.32\times A_{663}$$

(3) $$Cx=(1000\times A_{470}-2.05\times Ca-114.8\times Cb)/245$$

where A_663_, A_645_, and A_470_ represent the absorbance at 663, 645, and 470 nm, respectively.

#### 2.2.3. Determination of Turbidity

Appropriate modifications were made following the method of Kubo et al. [18]. Briefly, 5 g samples were centrifuged at 6000 r/min for 10 min at 20 °C, and the supernatant was measured at 660 nm using a spectrophotometer (Pulsar T9CS, China).

#### 2.2.4. Determination of Stability

The mass of the volumetric flask was recorded as m_1_. Subsequently, 5 g of well-mixed kiwifruit pulp was centrifuged at 6000 r/min for 10 min. The supernatant was then poured out and weighed, recorded as m_2_. The stability, represented as centrifugal sedimentation rate, was calculated using Equation (4).

(4) $$Stability\;\left(\%\right)=\frac{m_{2}-m_{1}}{5}\times 100\%$$

where m_1_ is the mass of the centrifuge tube; m_2_ is the total mass of the centrifuge tube and the precipitate.

#### 2.2.5. Determination of pH

A pH meter (METTLER, FE28-Standard, Zurich, Switzerland) was turned on and allowed to warm up for 15 min. It was then calibrated with a calibration solution. The probe was inserted into the pulp, and the readings was recorded after the values stabilized.

#### 2.2.6. Determination of Viscosity

The kiwifruit pulp was contained in a 600 mL beaker, ensuring it was free of air bubbles. The viscometer (DV3TLVTJ0 150 VA, Middleboro, MA, USA) was positioned horizontally on the tabletop, maintaining a constant temperature at 25 °C. A suitable rotor (LV-3) was selected and placed in the center of the pulp. The parameters were adjusted to a rotational speed of 120 r/min and a duration of 8 min, with data collection every 30 s. The data was recorded simultaneously at these intervals and subsequently transferred to the rotor for analysis.

### 2.3. Determination of Antioxidant Activity in Fruits

#### 2.3.1. Determination of DPPH and ABTS Free Radical Scavenging Rates

DPPH and ABTS free radical scavenging rates (DPPH-SR and ABTS-SR) were measured using previous methods [19]. Fruit pulp (4 g) was diluted 10 times and centrifuged at 8000 r/min for 10 min. The supernatant was collected, and an equal volume of ethanol was mixed with the DPPH solution at a 1:1 ratio, designated as A_0_. An equal volume of the sample and DPPH solution was aspirated, labeled as A_i_; and an equal volume of the sample and 95% ethanol was aspirated, recorded as A_j_. The reaction was conducted at room temperature, with the cuvette shielded from light for 30 min, after which the absorbance was measured at 517 nm using a spectrophotometer (Pulsar T9CS, China) and calculated using Equation (5).

(5) $$\mathrm{DPPH}-\mathrm{SR}\;\left(\%\right)=\left(1-\frac{A_{i}-A_{j}}{A_{0}}\right)\times \;100\%$$

ABTS mother liquor was prepared by mixing a 7.4 mmol/L ABTS solution with a 2.6 mmol/L potassium persulfate solution at a 1:1 ratio, followed by reacting in the dark for 12 h. The absorbance was then diluted with ethanol to achieve a final absorbance of 0.70 ± 0.02 at 734 nm. Next, 4 mL of the ABTS mother liquor and 1 mL of 95% ethanol were mixed thoroughly and recorded as A_n_. Subsequently, 4 mL of the ABTS mother liquor and 1 mL of the sample were mixed well, and recorded as A. After reacting for 6 min in the dark, the mixture was measured at 734 nm, and calculated using Equation (6).

(6) $$\mathrm{ABTS}-\mathrm{SR}\;\left(\%\right)=\frac{A_{n}-A}{A_{n}}\times 100\%$$

#### 2.3.2. Determination of Malondialdehyde (MDA)

MDA is the hallmark product of lipid peroxidation in fruits and vegetables, reflecting the degree of oxidation of cell membrane lipids. MDA content was determined using the thiobarbituric acid (TBA) method [20]. Approximately 1 g of kiwifruit pulp was mixed with 5 mL of 100 g/L trichloroacetic acid and centrifuged at 10,000× *g* for 20 min at 4 °C. Then, 2 mL of supernatant was combined with 2 mL of 0.67% TBA, boiled for 20 min, and then centrifuged, followed by measurement at 450, 532, and 600 nm using a spectrophotometer (Pulsar T9CS, China).

#### 2.3.3. Determination of Total Phenols

The total phenol content (TPC) was determined using the Folin–Ciocalteu method [21]. First, 2 mL of fruit pulp was added into 8 mL of pre-cooled ethanol and underwent ultrasonic extraction for 15 min. Subsequently, the mixture was centrifuged at 9000 r/min and 4 °C for 15 min. Then, 5.8 mL of distilled water, 0.5 mL of Folin–Ciocalteu reagent and 2 mL of 20% Na_2_CO_3_ were added. Finally, the mixture was diluted to a total volume of 10 mL with distilled water. After thoroughly mixing, it was placed in a light-proof environment for 1 h, followed by measurement at 760 nm using a spectrophotometer (Pulsar T9CS, China).

#### 2.3.4. Determination of Total Flavonoids

The determination was conducted following the method of Yuan et al., with appropriate modifications [22]. Kiwifruit pulp was placed in a drying oven for 5 h, after which it was weighed, and ethanol was added in a specific ratio. The mixture was then centrifuged at 4000 r/min for 10 min to obtain the supernatant of the crude flavonoid extract. The absorbance was measured at 510 nm using a spectrophotometer (Pulsar T9CS, China), and the total flavonoid content (TFC) was calculated using Equation (7).

(7) $$TFC\;(mg/100\;g)=\frac{C\times V\times N}{W}$$

where C is the concentration of flavonoids, mg/mL; V is the total volume of sample extract, mL; N is the dilution factor of the sample extract; W is the weight of the sample, g.

#### 2.3.5. Determination of Vc

The Vc assay was performed using the 2, 6-dichloroindifol titration method [23].

### 2.4. Determination of Fruit Flavor

#### 2.4.1. Determination of Total Soluble Solids (TSS)

Kiwifruit pulp was homogenized and the TSS was determined using a hand-held digital refractometer (LICHEN, Xiamen, China). The instrument was calibrated to 0° Brix with distilled water and a drop of pulp was placed on the prism surface and the cover plate was closed to ensure that there were no air bubbles before reading. Three parallel measurements were made for each group of 10 fruits and the results were averaged.

#### 2.4.2. Determination of Total Acid (TA)

After homogenization, kiwifruit pulp was used to determine TA by acid–base titration. Each group of 10 fruits was weighed accurately with 5.00 g of pulp, diluted with 50 mL of distilled water, and filtered after magnetic stirring for 5 min. Briefly, 10 mL of filtrate mixed with 2 drops of 1% phenolphthalein indicator was titrated with 0.05 mol/L NaOH standard solution to the end point (reddish color and does not fade for 30 s). A blank test was also carried out for correction. TA content was calculated according to Equation (8).

(8) $$TA\;(\%)=\frac{V\times C\times K\times D}{m\times 1000}\times 100$$

where V is the volume of NaOH solution, mL; C is the concentration of NaOH, mol/L; K is the conversion factor for organic acids (0.067 for malic acid); D is the dilution factor; m is the mass of the sample, g.

#### 2.4.3. Determination of Sugar/Acid Ratio

The sugar/acid ratio was determined using a kiwifruit-specific sugar/acid ratio analyzer (AITUO, PAL-1, Tokyo, Japan). Each group of 10 fruits was homogenized and 0.5 mL of homogenate was injected directly into the sample cell of the instrument. The instrument is equipped with a built-in high-precision refractive sensor and a pH/TA electrode, which can automatically complete the simultaneous detection of sugar and acid and output the sugar/acid ratio value.

#### 2.4.4. Electronic-Nose (E-Nose) Analysis

An E-nose instrument (SuperNose, Isenso, New York, NY, USA) was utilized for the analysis [23]. A total of 4 mL of fruit pulp was aspirated into a 15 mL headspace vial. The initial temperature of the trap was set at 40 °C and incubated at 60 °C for 20 min. The injection volume was 3 mL, with a washing time of 90 s. The filling rate was 500 μL/s, and the injection rate was 125 μL/s. The temperature of the inlet port was maintained at 200 °C, with an inlet pressure of 10 kPa. The outlet flow rate was 30 mL/min, and the injection time lasted for 45 s, followed by a washing time of 200 s. Data acquisition occurred over a period of 120 s, and each sample was analyzed in triplicate. The data was processed using Alpha Soft.

#### 2.4.5. Electronic Tongue (E-Tongue) Analysis

An E-tongue is capable of digitizing taste information and providing a more detailed analysis of flavor changes [24]. After powering on the instrument (SmarTongue, ISENSO, New York, NY, USA) for a 1 h warm-up equilibrium and performing instrument self-calibration, the acquisition temperature was set to 25 °C. The sensor was cleaned with distilled water as a cleaning solution prior to sampling. Data collection was performed by placing 10 mL of pulp sample into a standard 20 mL beaker of the E-tongue, with the probe inserted vertically below the liquid surface. The data were analyzed using Alpha Soft.

#### 2.4.6. Determination of Aroma Compounds

The composition of aroma compounds was analyzed using a headspace solid-phase microextraction (HS-SPME) and gas chromatography–mass spectrometry device (GC-MS) (Shimadzu, GCMS-QP2010SE SYSTEM, Kyoto, Japan) [25]. The pulp (5 g) was added into a 20 mL extraction bottle with 10 μL of 3-octanol at a concentration of 50 mg/mL as an internal standard. Then it was incubated at 40 °C for 40 min. A 50/30 μm DVB/CAR/PDMS (Divinylbenzene/Carboxen/Polydimethylsiloxane) extraction head was used to identify and adsorb aroma compounds.

GC conditions: a DB-5MS capillary column (30 m × 0.25 mm, 0.25 μm) was used; the carrier gas was helium (99.999%) at a flow rate of 1.0 mL/min; and constant flow was splitless injection. The injection port temperature was 220 °C, and the initial column temperature was 40 °C. Temperature program was conducted as follows: hold at 40 °C for 2 min; increase to 130 °C at a rate of 3 °C/min and hold for 2 min; and increase to 220 °C at a rate of 5 °C/min and hold for 5 min. MS conditions: ion source temperature was 200 °C; interface temperature was 230 °C; electron energy was 70 eV; and mass scanning range (*m*/*z*) was 45–270.

#### 2.4.7. Qualitative Quantification of Volatile Organic Compounds (VOCs)

Qualitative analysis: The raw data was analyzed using an Agilent MassHunter (Shimadzu, GCMS-QP2010SE SYSTEM, Kyoto, Japan). Signals with aligned quantitative and qualitative ion peaks were detected and subsequently annotated based on the MS2T database [26].

Quantitative analysis: MassHunter quantitative software was utilized to integrate and calibrate the peaks of the mass spectrometer. Subsequently, the peak areas were normalized and combined with the concentrations of internal standard substances to further quantify the VOCs in the sample. The VOC content in kiwifruit pulp was calculated using Equation (9) [27,28]. The quantitative data was standardized based on the isotope internal standard.

(9) $$Nx=\frac{Sx\times No}{So}$$

where *Nx* is the content of VOCs; *Sx* is the peak area of VOCs; *No* is the content of the internal standard substance; and *So* is the peak area of the internal standard material.

### 2.5. Statistical Analysis

Three parallel treatments were performed for the determination of each method, and the experimental data was analyzed using one-way analysis of variance (ANOVA) and expressed as mean ± standard deviation. Duncan’s multiple range test was employed to assess significant differences between means at the 95% confidence level. All statistical analyses were conducted using SPSS software (version 22; SPSS Inc., Chicago, IL, USA). GraphPad Prism (version 10.4.1) and RStudio (version 31.7.7) were utilized for graphing.

## 3. Results and Discussion

### 3.1. Texture and Color Variation of the Fruit

**Figure 1:**
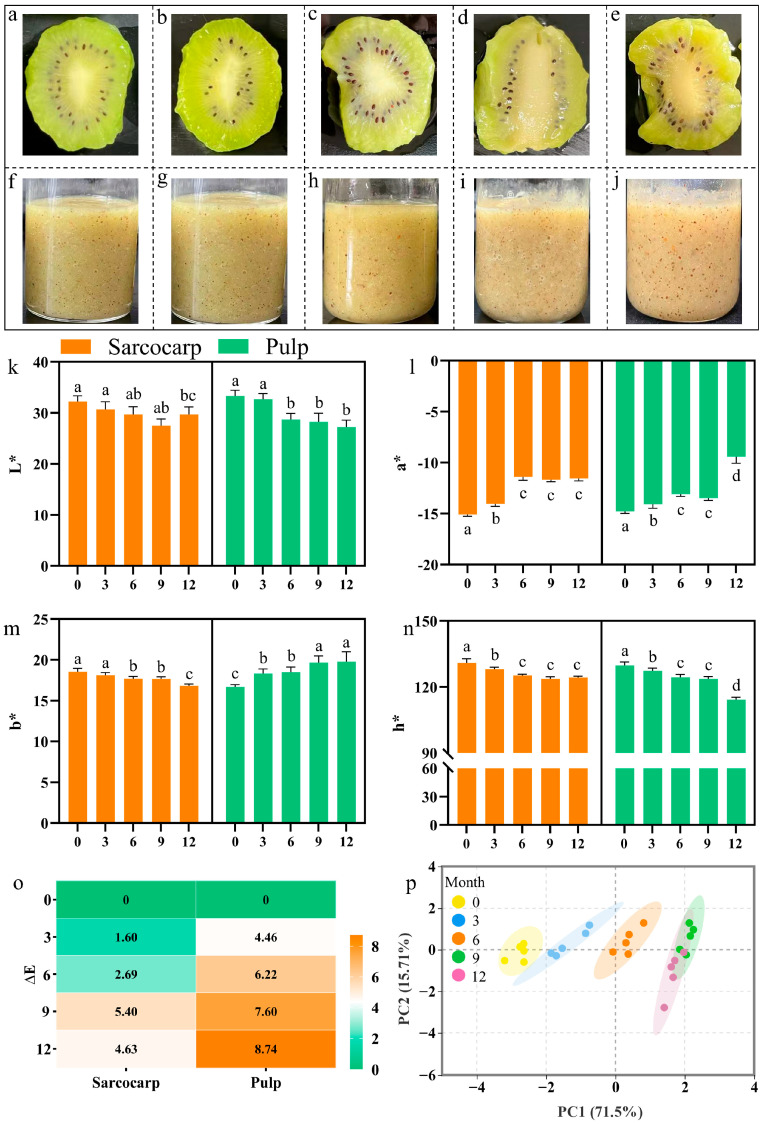
Characterization and color variation of kiwifruit sarcocarp and pulp during frozen storage. (**a**–**e**) Changes in sarcocarp phenotype after 0, 3, 6, 9, and 12 months of frozen storage; (**f**–**j**) changes in pulp color after 0, 3, 6, 9, and 12 months of frozen storage; (**k**–**o**) changes in L*, a*, b*, h*, and ΔE of kiwifruit sarcocarp and pulp after 0, 3, 6, 9, and 12 months of frozen storage. (**p**) PCA analysis of color changes in kiwifruit pulp after 0, 3, 6, 9, and 12 months of frozen storage. Values with different letters are significantly different (*p* < 0.05).

Phenotype is a crucial characteristic of frozen fruit products. The crystallization process that occurs during frozen storage causes the fruit to expand [29]. Color serves as an important evaluation criterion for consumers when making purchasing decisions [30,31]. After frozen storage, the phenotype of kiwifruit flesh changed significantly upon thawing (Figure 1a–e). After 3 months of freezing (Figure 1a,b), the flesh and core of the fruit remained relatively intact and preserved a complete oval shape. The juice was tightly encapsulated within the cellular structure and tissue, with no noticeable seepage. However, the edges of the slices exhibited slight wrinkling due to the formation of ice crystals. After 6 months, sensory deterioration accelerated. The pulp color began to darken and the L* value declined, and even the integrity of the slices decreased. Moreover, the a* value gradually increased and indicated a decrease in green, which was significantly different from the values observed after 3 months (*p* < 0.05). After 9 months of frozen storage, the pulp gradually loosened, and the amount of juice loss increased. Further dulling of the pulp and flesh color resulted in a significant difference in the b* value of the pulp compared to that at 6 months. The samples frozen for 12 months had lost their fundamental morphological characteristics. Upon thawing, they exhibited a mushy consistency, accompanied by significant juice loss, and increased turbidity resulting from damage to the pulp tissue. The h* value indicated a shift in color to an uneven brown-yellow hue. The total color difference in the pulp was greater than that in the sarcocarp, and the color difference value increased with the prolonged storage duration. This is in addition to being associated with a reduction in pulp tissue and an increase in the proportion of cores [32]. On the other hand, it is associated with the destruction of the cell membrane system by mechanical cuts during pulping, leading to changes in the distribution of enzymes and substrate regions in the cell membrane, which promotes the oxidation reaction of phenolic substances, and ultimately the brown substance [33]. In addition, the degradation of chlorophyll during long-term freezing is also an important cause [34] (Figure 1o). To fully understand the color changes caused by varying frozen durations, principal component analysis (PCA) was employed to characterize the color of kiwifruit pulp [35]. The results showed that the cumulative variance contribution rate of PC1 and PC2 reached 87.21% (Figure 1p), indicating that duration of freezing significantly influences the color change in kiwifruit pulp.

### 3.2. Changes in Functional Components and Physical Stability

**Figure 2:**
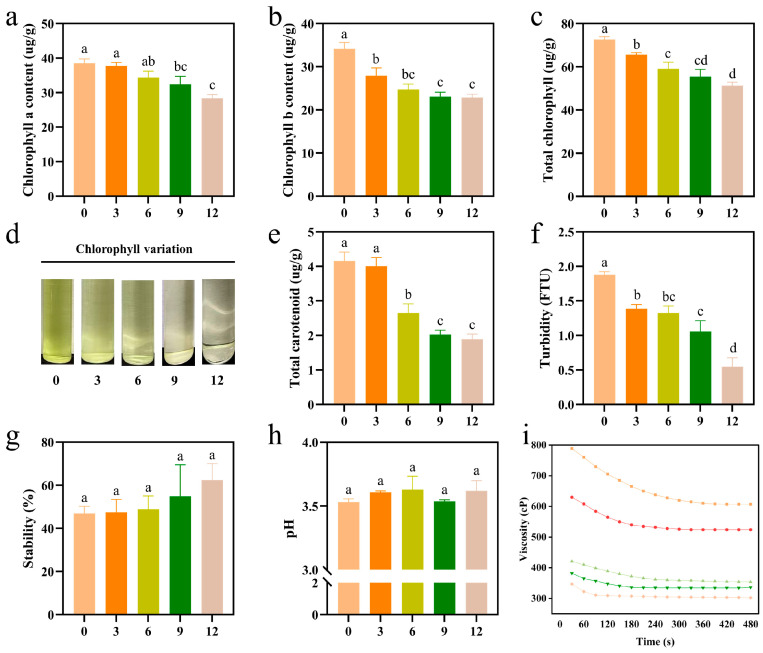
Changes in the quality of kiwifruit pulp during frozen storage. (**a**) Chlorophyll a content; (**b**) chlorophyll b content; (**c**) total chlorophyll content; (**d**) visualization of the content of chlorophyll extracted during 0, 3, 6, 9, and 12 months of frozen storage; (**e**) total carotenoid content; (**f**) turbidity; (**g**) stability; (**h**) pH; and (**i**) viscosity. Top to bottom lines is 0, 3, 6, 9 and 12 months of frozen storage. Values with different letters are significantly different (*p* < 0.05).

The functional component of fruit determines its processability. After being frozen for varying durations, the chlorophyll content of kiwifruit decreased. The chlorophyll a content was significantly different from that of fresh fruit by the ninth month, while the chlorophyll b content showed a significant difference by the sixth month. However, no significant differences were observed between the two as the freezing duration continued to increase (Figure 2a,b). The total chlorophyll content decreased by 29.45% over 12 months of frozen storage (Figure 2c), and the degradation process of chlorophyll is illustrated in Figure 2d. From 0 to 6 months, the extracts retained a bright green color; however, after 9 months, the loss of chlorophyll was so pronounced that the extracts appeared nearly transparent. Aglar et al. found that chlorophyll in kiwifruit slices decreased during cold storage and produced visual sensory changes [15]. Chlorophyll a and b decayed exponentially with time during freezing and that the rate of degradation was positively correlated with temperature [36,37]. More rapid freezing is considered the best method for preserving chlorophyll, such as liquid nitrogen freezing for short periods of time [38]. The stability of carotenoids in the freezing environment is influenced by several factors [39]. There was no significant change in carotenoid content after 3 months. However, after 6 months, its content decreased by 36.24% compared to the initial measurement at 0 months. Following an extended frozen storage of 9 and 12 months, the content further decreased by 51.26% and 54.43%, respectively (Figure 2e). The carotenoid content of kiwifruit varies between varieties and maturity stages during refrigeration. It has been shown that pre-harvest spraying with melatonin can promote the maintenance of chlorophyll and carotenoid content of kiwifruit during storage [40,41]. Turbidity is a crucial sensory and quality indicator in fruit juice products. Juices with higher turbidity levels contain more nutrients [42]. As illustrated in Figure 2f, after three months of frozen storage, the turbidity of the fruit significantly decreased, indicating that the ice crystals formed during freezing caused damage to the tissues and cells, resulting in the outflow of cellular fluid and the loss of certain nutrients. At progressively longer frozen durations, turbidity did not significantly decrease until the end of storage. The centrifugal sedimentation rate increased as turbidity decreased (Figure 2g). It suggests that the irreversible tissue damage to the kiwifruit caused by crystallization during frozen storage results in the loss of certain nutrients essential for processing, ultimately leading to a reduction in its stability. During the frozen storage, the pH of kiwifruit pulp did not change significantly (Figure 2h). Viscosity serves as an indicator of the pectin content in the pulp [22]. Our results demonstrate that the viscosity of kiwifruit pulp gradually decreases as the increasing duration of frozen storage, with a more pronounced decline at the six-month mark (Figure 2i). Hence, the fruit experiences slow oxidation during extended frozen storage, leading to the oxidation and breakdown of pectin molecules, which in turn reduces their molecular weight. Additionally, the damage caused by ice crystals to the cell structure leads to the loss of macromolecules such as pectin and proteins, which leach out with the precipitation of the juice. This process weakens the colloidal stability of the pulp, ultimately resulting in a decrease in viscosity.

### 3.3. Changes in Antioxidant Activity in Fruits

**Figure 3:**
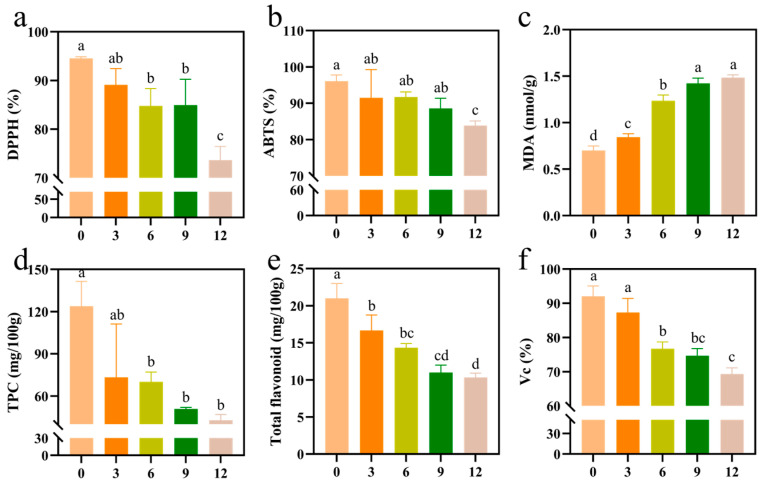
Changes in antioxidant activity of kiwifruit pulp during frozen storage. (**a**) DPPH radical scavenging; (**b**) ABTS radical scavenging; (**c**) MDA content; (**d**) TPC content; (**e**) total phenol content; and (**f**) Vc content. Values with different letters are significantly different (*p* < 0.05).

The antioxidant capacity of fruits not only influences their own storage tolerance but also is closely related to the development of functional products [43]. The DPPH free-radical scavenging rate after different storage durations decreased by 5.77%, 10.34%, 10.18%, and 22.13%, respectively. No significant difference in the DPPH free-radical scavenging rate was observed from 0 to 3 months; however, a significant decline was observed from the sixth month onwards. At the end of the storage, the DPPH free-radical scavenging rate decreased the most and was significantly different from the rates at other time points (Figure 3a). The change in the ABTS free-radical scavenging rate was like that in the DPPH free-radical scavenging rate. Over the storage period of 0 to 12 months, the ABTS free-radical scavenging rate decreased by 4.75%, 4.54%, 7.82%, and 12.7%, respectively. The 12-month treatment exhibited a significant difference compared to the other groups (Figure 3b). In addition to changes in phenolic, Vc, and carotenoid contents, the decrease in antioxidant properties associated with low-temperature stress was attributed to the impairment of cell membrane fluidity in kiwifruit pulp cells, leading to lipid peroxidation of the cell membranes and a decrease in the activity of antioxidant enzymes [39,44,45]. Damage to the membrane system is a common adverse effect observed in frozen fruit [46]. The content of MDA increased gradually during the frozen storage, with significant differences noted between the 0–9 months duration and the 9–12 months duration treatments (Figure 3c). It indicates that prolonged frozen storage severely compromises the integrity of pulp cell membranes. The total phenolic content in plants is regarded as one of the most effective molecules for neutralizing free radicals [47]. Kiwifruit exhibited a significant decrease in phenolic compounds starting from the pre-freeze storage period, with a 40.66% reduction observed in the 3 months. Since then, the phenolic levels remained consistently low, with no significant differences detected (Figure 3d). The total flavonoid content exhibited a continuous downward trend throughout the frozen storage period (Figure 3e). After 12 months of storage, it decreased by 50.95%. This decline may be attributed to the formation of ice crystals during the frozen storage, which disrupts the cellular structure and allow phenolic substrates to meet polyphenol oxidase. The observed change in color difference indicates that this interaction triggers the corresponding enzymatic browning reaction [48]. Furthermore, the frozen storage may alter the stability of the glycosidic bonds in flavonoid compounds by breaking hydrogen bonds, thereby accelerating their degradation [49]. Vc is a crucial antioxidant found in kiwifruit and plays a significant role in abiotic stress responses and reactive oxygen species metabolism [50]. Our study revealed that after three months, the Vc content decreased with no significant difference (Figure 3f). This may be attributed to the fact that cold stress stimulates the synthesis of Vc, thereby mitigating oxidative damage to the fruit caused by low temperatures [51]. By six months, the Vc content began to decline significantly, indicating that the antioxidant metabolism system in kiwifruit became disordered. Ultimately, the Vc content stabilized at approximately 69.5%, which was nearly 30% lower than that of fresh fruit. Flavonoids, a prominent group of phenolic compounds, are widely distributed in plants and serve as important indicators of antioxidant activity [52].

### 3.4. Changes in Key Flavor Determinants and Overall Flavor Perception

**Figure 4:**
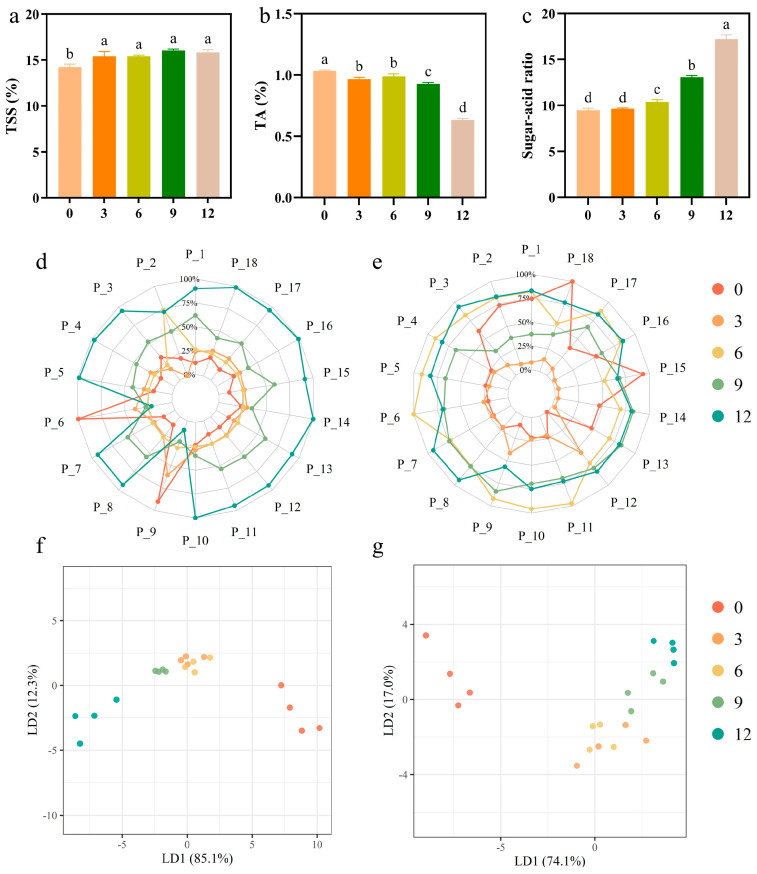
Flavor-related characteristics of kiwifruit pulp during frozen storage. (**a**) TSS content; (**b**) TA; (**c**) sugar/acid ratio; (**d**) E-nose analysis (aroma profile); (**e**) E-tongue analysis (taste profile); (**f**) E-nose LDA analysis; (**g**) E-tongue LDA analysis. Values with different letters are significantly different (*p* < 0.05).

Fruit pulp serves as a raw material for further processed products, including beverages and jams, and its flavor stability is a critical evaluation criterion. Variations in flavor precursors, such as TSS, TA, and sugar/acid ratio, during frozen storage can significantly impact the potential for the widespread application of fruit pulp in product manufacturing [53]. As illustrated in Figure 4a–c, TSS exhibited a slight increase during the frozen storage, with significant differences observed between 0 and 3 months. However, TSS changed only marginally from 3 to 12 months, with no significant differences noted. Research indicates that freezing can inhibit amylase activity, but prolonged exposure to low temperatures can lead to structural damage in cells, causing starch to gradually degrade into soluble sugars [54]. These result in minor fluctuations in TSS, although overall stability is maintained [55]. Similarly, the integrity of the pulp cell membrane is compromised, leading to the loss of acidic substances through the exudation of cell fluid [56]. Figure 4b showed that the change in TA content was dramatically pronounced, exhibiting a downward trend and an accelerated decline rate over time. After 12 months, the accumulation of degradation products may reach a threshold, leading to the maximum reduction in TA [10]. Concurrently, certain acids may be converted into sugars via the gluconeogenesis pathway. For instance, malic acid can undergo decarboxylation to produce pyruvic acid, which is involved in sugar metabolism and indirectly contributes to an increase in TSS content [57]. Figure 4c illustrates that the sugar/acid ratio of the pulp increased significantly with storage time. The notable decrease in TA was the primary factor contributing to the rise in the sugar/acid ratio. Hence, when the fruit sweetness is enhanced, the acidity becomes unbalanced and adversely affects the overall flavor [58].

An E-nose can detect complex odors and volatile components in kiwifruit pulp. These parameters serve as important indicators for determining the flavor of fruit pulp [59]. Figure 4d illustrates the radar graph depicting the E-nose’s response during various frozen storage. The differences observed in the radar graph reflected the intensity of the fruit pulp odor to some extent (the significance of the sensor is detailed in Table S1). All 18 sensors responded to the pulp during varying periods and were able to distinguish the pulp at different frozen durations. In the present study, the sensor response intensity was low after 3 and 6 months of frozen storage, with the radar graphs nearly overlapping. At 9 months, each sensor exhibited a strong response, reaching a peak at the end of frozen storage. Among the 18 sensors, P_6 and P_9 exhibited the strongest response intensity to the pulp at the beginning of storage, indicating the flavor intensity of aromatic compounds, alcohols, and aldehydes during the early stages. The E-tongue effectively eliminates subjective factors in the detection of taste characteristics. As illustrated in Figure 4e, sensors P_15 and P_18 displayed a high response intensity at the beginning of storage, followed by a decreasing trend as storage duration extended. Other sensors demonstrated significant variations during each stage, suggesting a signification change in the taste quality of fruit pulp.

Linear discriminant analysis (LDA) is a method used in cluster analysis that emphasizes the distance analysis among different groups, allowing for hierarchical judgments regarding these groups [60]. The E-nose analysis indicated that LD1 and LD2 accounted for 85.1% and 12.3% of the variance, respectively, resulting in a cumulative total of 97.4% (Figure 4f). The fruit pulp exhibited significant differences due to the varying frozen durations, with the most pronounced disparity observed between the samples stored for 0 months and those stored for 12 months. In contrast, the samples stored for 3 to 9 months did not show significant separation, which aligned with the findings from the E-nose odor assessment. The LDA conducted with the E-tongue (Figure 4g) revealed that LD1 (74.1%) and LD2 (17%) accounted for 91.1% of the variance. Unlike the results of the E-nose, the E-tongue analysis indicated that the 0-month sample was distinctly separated from the other groups. However, the samples could not be effectively distinguished after prolonged duration, suggesting that frozen duration significantly impacts the taste characteristics.

### 3.5. Changes in VOCs in Fruits

**Figure 5:**
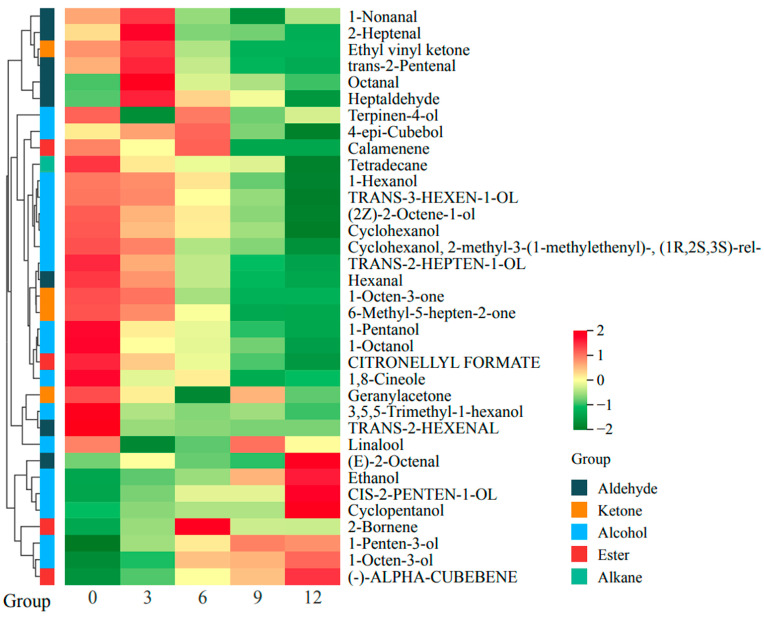
Heat map and hierarchical cluster analysis of kiwifruit during frozen storage.

Aroma is a crucial factor influencing the quality of kiwifruit pulp and the preferences of consumers. Figure 5 illustrates the changes in volatile organic compounds of kiwifruit pulp after frozen storage. Specific content changes are detailed in Table S2. The concentration of aroma compounds indicates their response intensity [61]. Following prolonged frozen storage, HS-SPME-GC-MS was employed to identify a total of 35 volatile compounds that represent the aroma components. Nineteen VOCs, including tetradecane, trans-3-hexen-1-ol, 6-methyl-5-hepten-2-one, and trans-2-hexenal, exhibited higher abundances during early storage (≤3 months). These compounds impart fresh grassy and citrus notes, and this result is consistent with the findings of Wang et al. that immature kiwifruits retain herbaceous aromas, which are later masked by fruity esters during ripening [62]. Five aldehydes (1-nonanal, 2-heptenal, trans-2-pentenal, octanal, and heptaldehyde) and one ketone (ethyl vinyl ketone) peaked after 3 months, followed by a progressive decline. Furthermore, 2-bornene, a pine-resin-scented ester, displayed an initial increase followed by a reduction, which is consistent with prior reports on ester volatility under freezing stress [63]. By 12 months, fruity esters diminished markedly, while compounds such as (E)-2-octenal, cis-2-penten-1-ol, and (-)-alpha-cubebene accumulated, contributing fatty, fermented, and earthy off-odors. Ethanol levels surged after 9 months, suggesting potential microbial fermentation that could be detrimental to pulp quality.

The results reveal that long-term frozen storage affects the changes in the volatile components of the fruit flesh, thereby influencing the flavor of fruit pulp. Monitoring the aroma compounds of kiwifruit pulp can help determine the optimal frozen storage duration for kiwifruit. Based on this information, by-products can be processed more effectively to maximize fruit utilization and align with consumer preferences.

### 3.6. Correlation Analysis

**Figure 6:**
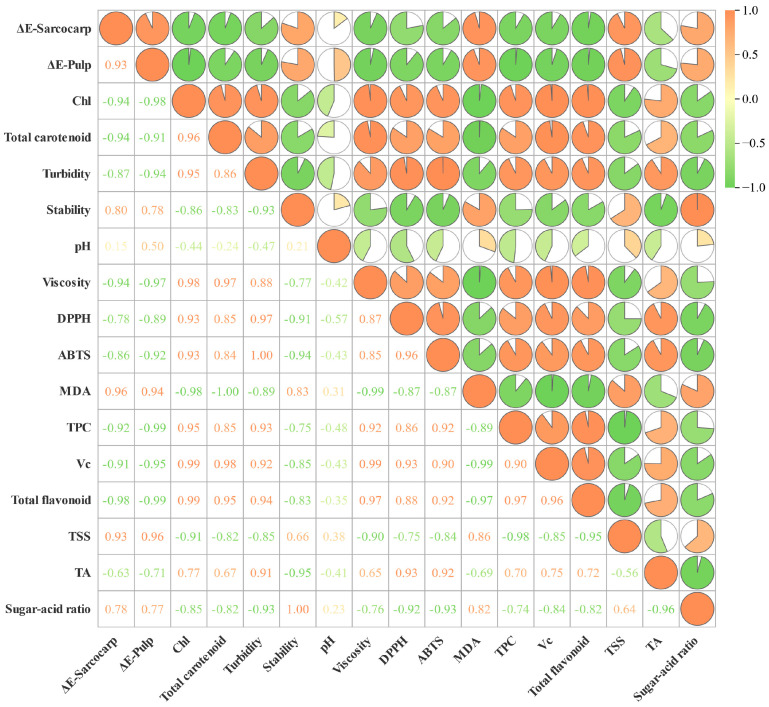
Correlation distribution between color difference (sarcocarp and pulp), quality, antioxidant activity, and flavor indices of kiwifruit. Red marks a positive correlation, while green marks a negative correlation. The higher the correlation, the larger the sector area. The numerical value represents Pearson’s correlation coefficient; |r| > 0.9 is considered highly correlated.

To better understand the correlation between the color differences (pulp and peel), pulp quality, antioxidant activity, and flavor index of ‘Xuxiang’ kiwifruit after long-term frozen storage, correlation analysis was employed to elucidate the results (Figure 6). Long-term frozen storage significantly affects the dynamic balance of metabolites and the antioxidant network in kiwifruit. TSS is strongly correlated with the color difference values of both pulp and peel, which may be linked to the activation of the starch–sugar conversion pathway induced by low temperatures [64]. Studies have shown that the upregulation of sucrose phosphate synthase gene expression in this process accelerates the degradation of chlorophyll mediated by sugar metabolites, resulting in a negative correlation between chlorophyll and carotenoids. Additionally, with the starch hydrolysis, TA decreases, which may be attributed to the conversion of organic acids into sugars and their derivatives during the freezing process, as well as their utilization in respiration [65]. The free-radical scavenging ability of DPPH and other substances, along with the synergistic antioxidant effects of Vc and TPC during frozen storage, collectively preserves the quality of fruit pulp. This confirms the synergistic antioxidant pathway of phenolic compounds through the hydroxyl donor mechanism and the Vc regeneration cycle [14]. MDA is a marker for lipid peroxidation, and its accumulation reflects the quality of the fruit pulp. An increase in MDA indicates that oxidative stress is exacerbated, leading to damage to the integrity of the cell membrane and significant cytoplasmic leakage. This results in a decrease in turbidity, viscosity, and stability of the fruit pulp [66,67]. The sugar/acid ratio is regarded as positively correlated with flavor retention. In this study, a strong positive correlation between the sugar/acid ratio and the stability of the fruit pulp was observed, and this aligns with the conclusion of Jiang et al., which suggests that the sugar/acid ratio can indirectly reflect the maintenance of cellular integrity [68]. In addition, the sugar/acid ratio serves several functions, including inhibiting microorganisms during fruit storage, synergistically enhancing the activity of antioxidants, maintaining the sweet–acid balance of the pulp, and extending shelf life. It acts as a comprehensive biomarker [69]. The combination of kiwifruit quality indicators, antioxidants, and volatile compounds offers a thorough overview of changes during long-term frozen storage. Herein, it systematically analyzes the challenges encountered by kiwifruit pulp during frozen storage, providing valuable insights for the efficient and rational use of pulp in practical applications. However, the exploration of other varieties as well as research into more advanced preservation techniques (e.g., air-conditioned dynamic monitoring, ultrasonic pretreatment, and ozone treatment) will be critical in the long-term processing and utilization of fruit pulp. Future research should link lab-scale efficacy with commercial scalability to advance the highly integrated utilization of the global fruit supply chain.

## 4. Conclusions

This study provides the first comprehensive assessment of long-term frozen viability and quality change in ‘Xuxiang’ kiwifruit during 12 months at −20 °C. In addition to confirming phenotypic deterioration and pigment degradation (e.g., >50% carotenoid decline), we demonstrated that key industrial attributes, including TSS, TA, and sugar/acid ratios, remained virtually stable throughout the 12-month storage. Critically, flavor deterioration became irreversible after 9 months and characterized by objectionable fat/fermentation volatiles. These findings establish a practical framework for the industry: despite quality changes, frozen pulp stored for ≤9 months retains acceptable level for premium fresh-cut and whole-fruit applications such as juices, purees, and functional ingredients. By linking fundamental quality decay mechanisms to tangible supply chain solutions, this work enables manufacturers to optimize frozen inventory turns, enable year-round kiwifruit processing, and develop targeted anti-freeze technologies that ultimately turn seasonal constraints into business opportunities.

## Data Availability

The original contributions presented in this study are included in the article/Supplementary Materials. Further inquiries can be directed to the corresponding authors.

## Supplementary Materials

The following supporting information can be downloaded at: https://www.mdpi.com/article/10.3390/foods14132322/s1, Table S1: Responders for electronic nose sensors; Table S2: Changes in relative contents of characteristic VOCs in kiwifruit.
