# Supplementary material for: Deterioration in the Quality of ‘Xuxiang’ Kiwifruit Pulp Caused by Frozen Storage: An Integrated Analysis Based on Phenotype, Color, Antioxidant Activity, and Flavor Compounds
Source: Foods. 2025 Jun 30;14(13):2322. doi: 10.3390/foods14132322 (PMC12249429; doi:10.3390/foods14132322)
Supplement: Supplementary file 1 [file foods-14-02322-s001.zip › foods-3692360-supplementary.pdf]

Table S1 Responders for electronic nose sensors

Sensors	Responsive substance	Substance type
Sn_1	Alkanes, smoke	Propane, natural gas, smoke
Sn_2	Alcohols, aldehydes, short-chain alkanes	Alcohol, smoke, isobutane, formaldehyde
Sn_3	Ozone	Hydrogen sulfide
Sn_4	Sulfide	
Sn_5	Organic amine	Ammonia, methylamine, ethanolamine
Sn_6	Organic gases, benzophenones, alkyds, aromatic compounds	Toluene, acetone, ethanol, hydrogen, other organic vapors
Sn_7	Short chain alkanes	Methane, natural gas, biogas
Sn_8	Short chain alkanes	Propane, LPG
Sn_9	Aromatic compounds, alcohols, and aldehydes	Toluene, formaldehyde, benzene, alcohol, acetone
Sn_10	Hydrogen-containing gas	Hydrogen
Sn_11	Alkanes, Olefins	Liquefied gas, alkanes, olefins

Sn_12	Short chain alkanes	Liquefied gas, methane
Sn_13	Combustible gas	Methane
Sn_14	Combustible gas	Combustible gas, smoke
Sn_15	Alkanes, organic gases	Smoke, isobutane, organic acid esters, aliphatic hydrocarbons
Sn_16	Sulfide	Sulfur compounds
Sn_17	Nitride	Nitrogen oxides
Sn_18	Ketones, alcohols	Acetone, ethanol, organic solvents

Table S2 Changes in relative contents of characteristic VOCs in kiwifruit

Number	Compounds	CAS	Content / (ug/L)				
			0	3	6	9	12
			Alcohol				
1	Ethanol	64-17-5	6.69±1.21	7.63±1.42	8.13±0.89	10.09±1.57	11.86±1.45
2	Cyclopentanol	96-41-3	5.88±0.74	7.75±1.01	8.56±1.71	8.56±1.41	19.89±3.21
3	1-Penten-3-ol	616-25-1	0.64±0.11	1.54±0.42	1.97±0.84	2.43±0.78	2.37±0.71
4	1,8-Cineole	470-82-6	53.54±7.45	38.31±4.52	40.86±5.14	29.29±3.54	31.37±4.20
5	1-Pentanol	71-41-0	38.12±4.65	31.30±5.47	30.06±6.54	26.36±4.14	24.77±3.59
6	CIS-2-PENTEN-1-OL	1576-95-0	0.71±0.21	1.14±0.24	1.50±0.32	1.50±0.38	2.80±1.01
7	1-Hexanol	111-27-3	30.64±6.51	29.43±8.54	24.53±4.21	16.52±3.90	8.14±2.52
8	TRANS-3-HEXEN-1-OL	544-12-7	1.12±0.26	1.07±0.44	0.74±0.16	0.51±0.11	-
9	Cyclohexanol	108-93-0	50.62±17.52	40.90±8.51	36.23±7.16	26.83±8.22	7.62±4.23
10	1-Octen-3-ol	3391-86-4	3.31±0.89	4.82±1.26	7.89±1.39	8.09±1.78	9.19±2.56
11	TRANS-2-HEPTEN-1-OL	33467-76-4	5.98±2.89	4.15±2.10	2.11±0.58	0.74±0.19	-

12	3,5,5-Trimethyl-1-hexanol	56114-69-3	9.62±2.34	1.62±0.25	0.97±0.08	1.34±0.89	-
13	4-epi-Cubebol	38230-60-3	0.80±0.07	1.00±0.24	1.16±0.44	0.46±0.04	-
14	Linalool	78-70-6	2.55±0.84	1.74±0.65	2.02±0.74	2.59±0.51	2.29±1.11
15	1-Octanol	111-87-5	8.33±2.95	5.42±2.21	5.13±1.74	4.14±1.34	2.91±1.31
16	Terpinen-4-ol	562-74-3	1.35±0.41	1.00±0.04	1.33±0.09	1.12±0.24	1.18±0.31
17	(2Z)-2-Octene-1-ol	26001-58-1	1.98±0.48	1.58±0.24	1.33±0.51	0.82±0.08	-
18	Cyclohexanol,2-methyl-3-(1-methylethenyl)-, (1R,2S,3S)-rel-	54244-81-4	1.28±0.07	1.13±0.20	0.54±0.06	0.44±0.03	-
Ketone							
1	Ethylvinylketone	1629-58-9	5.39±2.21	7.10±2.11	2.07±0.24	-	-
2	1-Octen-3-one	4312-99-6	1.64±0.21	1.50±0.57	0.47±0.26	-	-
3	6-Methyl-5-hepten-2-one	110-93-0	29.97±6.21	28.27±5.12	24.50±4.26	18.97±7.22	18.89±4.89
4	Geranylacetone	689-67-8	1.81±0.25	1.55±0.64	1.11±0.61	1.65±0.09	1.33±0.14
Aldeh							

yde							
1	Hexanal	66-25-1	20.27±3.62	15.89±5.21	8.01±4.12	2.72±0.84	0.96±0.07
2	trans-2-Pentenal	1576-87-0	2.91±0.52	4.10±1.01	1.68±1.42	0.67±0.05	0.48±0.06
3	Heptaldehyde	111-71-7	0.70±0.21	3.11±0.99	1.90±0.44	1.47±1.24	-
4	TRANS-2-HEXENAL	6728-26-3	53.54±18.66	2.64±0.22	1.38±0.09	-	-
5	Octanal	124-13-0	0.99±0.08	3.83±1.31	1.68±0.77	1.42±0.45	0.97±0.18
6	2-Heptenal	57266-86-1	6.51±2.33	9.46±2.14	4.59±1.28	4.48±1.22	3.48±0.59
7	1-Nonanal	124-19-6	5.12±1.26	6.17±3.21	3.38±0.91	1.87±0.68	3.55±0.92
8	(E)-2-Octenal	2548-87-0	1.10±0.55	2.56±0.62	0.98±0.41	0.61±0.07	6.49±1.52
Alkane							
1	Tetradecane	629-59-4	6.17±2.01	4.06±1.06	3.55±0.89	3.38±0.77	0.51±0.02
Ester							
1	CITRONELLYLFORMATE	105-85-1	7.76±2.69	5.12±2.01	3.94±1.31	2.13±0.88	0.56±0.07
2	(-)-ALPHA-CUBEBENE	17699-14-8	2.70±1.02	3.88±0.99	5.25±0.69	5.99±1.42	7.53±2.21

3	2-Bornene	464-17-5	0.80±0.0<br>8	0.89±0.15	1.16±0.57	0.92±0.41	0.92±0.07
4	Calamenene	483-77-2	0.82±0.0<br>9	0.50±0.22	0.90±0.16	-	-
